# Paradigm Shift in the Management of Acute Myeloid Leukemia—Approved Options in 2023

**DOI:** 10.3390/cancers15113002

**Published:** 2023-05-31

**Authors:** Naveen Premnath, Yazan F. Madanat

**Affiliations:** 1 Division of Hematology and Medical Oncology, Department of Internal Medicine, University of Texas Southwestern Medical Center, Dallas, TX 75235, USA; naveen.premnath@utsouthwestern.edu; 2 Leukemia Program, Harold C. Simmons Comprehensive Cancer Center, UT Southwestern Medical Center, Dallas, TX 75235, USA

**Keywords:** AML, chemotherapy, FDA approved, first line treatment, induction, 7 + 3, leukemia

## Abstract

**Simple Summary:**

Acute Myeloid Leukemia is the most common aggressive blood cancer in adults. Treatment for this condition is divided into induction followed by consolidation. The mainstay of treatment is chemotherapy, and until a decade ago, only traditional chemotherapy was approved for this condition. Induction involves treatment with intense chemotherapy for a span of 7 days. Once blood counts recover, patients undergo bone marrow biopsy to ensure remission status for the disease. Elderly patients who were considered too weak to receive intense therapy were treated with single-agent low-intensity therapy until the approval of a few new combinations starting in 2018. As a result, over the last 10 years, 10 new medications have been approved for the treatment of this type of leukemia. We discuss the evidence behind these treatment regimens and combinations.

**Abstract:**

The word Leukemia was coined nearly 200 years ago by Rudolf Virchow. Once a death sentence, Acute Myeloid Leukemia (AML) is now a treatable condition. The introduction of “7 + 3” chemotherapy, originally reported from the Roswell Park Memorial institute in Buffalo, New York, in 1973, changed the treatment paradigm for AML. About twenty-seven years later, FDA approved the first targeted agent, gemtuzumab, to be added to this backbone. During the last seven years, we have had ten new drugs approved for the management of patients with AML. Work by many dedicated scientists led to AML achieving the elite status of being the first cancer to have the whole genome sequenced using next-generation sequencing. In the year 2022, we witnessed the introduction of new classification systems for AML by the international consensus classification and the world health organization, both emphasizing molecular classification of the disease. In addition, the introduction of agents such as venetoclax and targeted therapies have changed the treatment paradigm in older patients ineligible for intensive therapy. In this review, we cover the rationale and evidence behind these regimens and provide insights into the newer agents.

## 1. Introduction

Acute Myeloid Leukemia (AML) is the most common acute leukemia among adults, with an estimated incidence of 20,050 new cases in 2022. AML is a disease of the older population with a median age of 68 years at diagnosis [1]. It is still a relatively rare malignancy that accounts for ~1% of all cancers in the United States, with an overall 5-year survival of 30.5% between 2012 to 2018 [1]. Acute myeloid Leukemia is, however, a heterogeneous disease with various genetic drivers that determine prognosis and disease outcomes. AML is a clonal disorder where the acquisition of somatic mutations in progenitor cells, provide selective advantage to these clones as a result of increased self-renewal with or without influence from environmental pressures [2]. The increased production of immature clones causes bone marrow failure due to impaired hematopoiesis. The ensuing anemia leads to symptoms of fatigue or shortness of breath, thrombocytopenia increases the risk of bleeding, and neutropenia manifests as fevers or infectious complications. We know that some of these mutations are acquired much earlier and often do not have any hematologic manifestations. When these mutations that are recurrently seen in myeloid malignancies are found at a variant allele frequency (VAF) ≥ 2% in the absence of cytopenia, the term clonal hematopoiesis of indeterminate potential has been coined [3]. However, other highly proliferative mutations such as *FLT3, RUNX1* and *RAS* are acquired towards the later stages of leukemogenesis and often present as second-hit mutations [4].

## 2. Changes in Disease Classification in 2022

**Figure 1:**
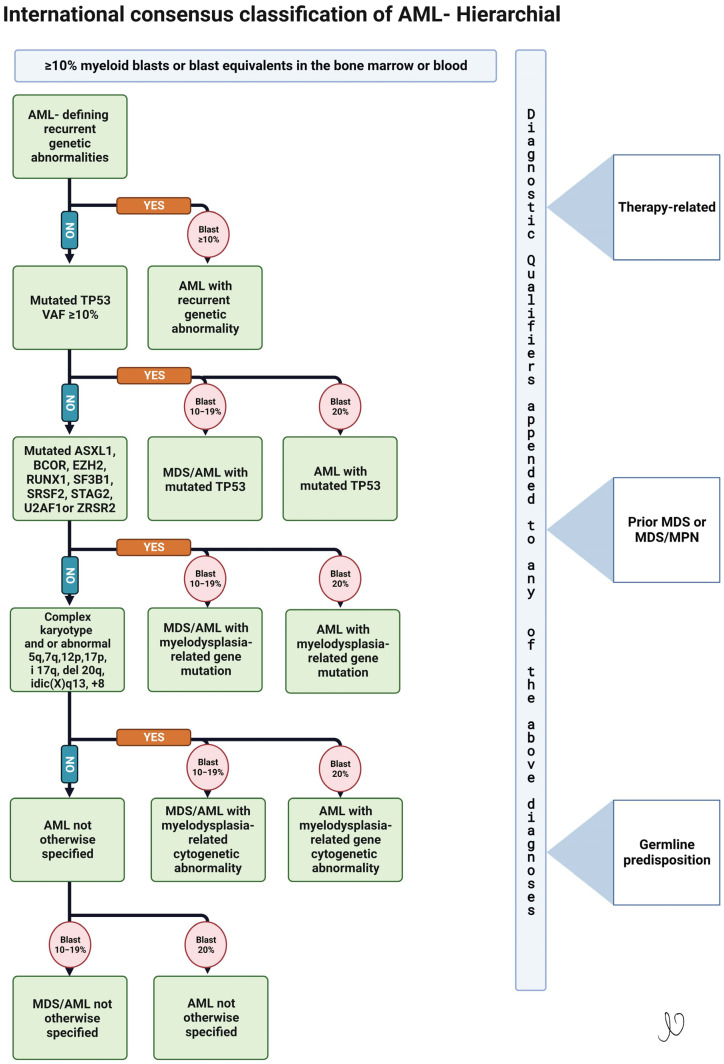
International Consensus Classification of AML—2022.

Recognizing the overarching importance of genomic and molecular characteristics of AML in determining treatment, prognosis and influence on future research, the international consensus classification (ICC) has revised and launched a new classification for AML in 2022 [5]. This new classification would now be a hierarchical classification, as guided by recent data that certain genomic and molecular characteristics have a stronger influence on prognosis than others. The World Health Organization (WHO) also published the 5th edition classification in 2022 [6]. Some key differences between the two classifications are that the ICC introduced a new entity of myelodysplastic syndrome (MDS)/Acute Myeloid Leukemia (AML) for patients with blast percentage of 10–19%, allowing those patients to potentially enroll in MDS or AML clinical trials. The WHO classification continues to recognize this entity as MDS with increased blast II. Given the significant influence seen with p53 mutations, ICC set forth a separate entity whenever *TP53* mutations are present, defined as AML with mutation *TP53* when a somatic *TP53* mutation is present with a variant allele frequency of >10%. However, a similar distinction was not seen with the WHO AML classification. Per ICC, there was a focus on molecular abnormalities which hold a higher significance with relevance to the disease classification; as such, therapy-related AML became a diagnostic qualifier, rather than its own entity, while the WHO created a separate new category called myeloid neoplasms post-cytotoxic therapy irrespective of the cytogenetics and or genomic abnormalities. The ICC allows a diagnosis of AML for patients with bone marrow or peripheral blood (PB) blasts ≥ 10% in addition to one of the following genetic abnormalities: t(15:17), t(8:21) and Inv 16, along with newly added abnormalities such as t(9:11), t(6:9), inv 3, mutated *NPM1* and in frame bZIP mutated *CEBPA*, and less common rearrangements in *RARA, MECOM* and *KMT2A.* AML with t(9:22) continues to require 20% blasts or more to distinguish it from accelerated phase CML. Per the WHO, a diagnosis of AML may be made for certain abnormalities irrespective of blast percent; these include *PML*::*RARA*, AML with *RUNX1*::*RUNX1T1* and AML with *CBF*::*MYH11*, in addition to mutations in *NPM1* and biallelic (biCEBPA) or single mutations located in the basic leucine zipper (bZIP) region of the gene (smbZIP-*CEBPA*). These are now AML-defining entities. Lastly, rearrangements involving *KMT2A, MECOM* and *NUP98* are added as genetic abnormalities that are acceptable to diagnose AML with <20% blasts; however, no specific blast cut-off requirement is specified. Figure 1 shows the new hierarchical classification by ICC.

## 3. Treatment of Newly Diagnosed AML

### 3.1. Standard Induction: 7 + 3 Chemotherapy

The standard of care for younger individuals with newly diagnosed AML has remained a combination of intensive chemotherapy that involves 7 days of cytarabine and 3 days of an anthracycline called the “7 + 3” for the past 40 years. However, multiple studies over decades have investigated better agents and looked at optimizing the doses; some of the studies are highlighted below.

### 3.2. Continuous vs. Intermittent Cytarabine

There were two major publications in Blood in 1996, first from the Australian study group by Bishop et al., who conducted a randomized trial with patients assigned to either continuous dose cytarabine 100 mg/m^2^ from day 1 to 7 vs. high dose cytarabine 3 g/m^2^ IV every 12 hours (h) on day 1, 3, 5 and 7, given along with Daunorubicin 50 mg/m^2^ day 1 to 3 and etoposide 75 mg/m^2^ day 1 to 7 in both arms. Post-remission therapy was the same for both groups and included continuous doses of cytarabine, daunorubicin and etoposide in a 5-2-5-day schedule, respectively [7]. The complete remission (CR) rates did not differ between the groups; 71% vs. 74%, with a *p* = 0.7. The second study was conducted by the Southwest Oncology Group (SWOG) and was published by Weick et al. [8] in October of the same year. Patients less than 65 years of age with newly diagnosed AML were randomized to either a standard dose of continuous cytarabine 200 mg/m^2^ on days 1 to 7 with daunorubicin 45 mg/m^2^ on days 5 to 7 vs. high dose cytarabine 2 g/m^2^ or 3 g/m^2^ given every 12 h from day 1 to 6, followed by daunorubicin 45 mg/m^2^ on day 7 to 9. The 3 g/m^2^ dose arm was soon discontinued due to increased neurological toxicities noted by the data monitoring committee. The final analysis included 665 patients, with 493 in the continuous group and 172 in the high dose, with no differences in CR rates (*p* = 0.96) or overall survival (OS) rates (*p* = 0.41). Later Kern et al. published a review of all three randomized studies to answer the above question and found no significant difference between the rates of complete remission (RR 1.00, *p* = 1), rate of early death (RR 1.53, *p* = 0.16) or median OS (*p* = 0.9). Of note, the high-dose cytarabine group showed an improved median recurrence-free survival (*p* = 0.03), the only finding that was common to all three studies as there was major inter-study variability for other end points [9].

Lowenberg et al. for the HOVON and SAKK group published data to prospectively address whether cytarabine dosing during induction chemotherapy impacts outcomes. Their study enrolled 860 patients and randomized patients into two arms: an intermediate dose of cytarabine (continuous dosing) and a high dose of cytarabine (intermittent higher dose). Patients randomized to the intermediate dose received idarubicin 12 mg/m^2^ for 3 days on days 5, 6 and 7 and continuous dose cytarabine 200 mg/m^2^ IV from days 1 to 7. Patients assigned to high-dose cytarabine received cytarabine 1 g/m^2^ twice daily dosing from days 1 to 5 in addition to the same idarubicin dose/duration. This was followed by a second induction cycle using a topoisomerase-II inhibitor and amsacrine, dosed at 120 mg/m^2^ IV on days 3, 5 and 7 in both arms in addition to cytarabine 1 g/m^2^ twice daily on days 1 to 6 in the intermediate dose cytarabine arm, vs. 2 g/m^2^ twice daily for eight doses on days 1, 2, 4 and 6 in the high dose cytarabine arm. There were no differences in the rates of complete remission after cycle 1 (60% vs. 66%) or cycle 2 (19% vs. 16%) *p* = 0.45, or in event-free survival (EFS) *p* = 0.79 or (OS) *p* = 0.87 [10]. Notably, there were signals of increased treatment deaths with the high-dose cytarabine protocol with an HR of 1.41 and (*p* = 0.06). Willemze et al. used a backbone of daunorubicin and etoposide for induction, combined with either a continuous cytarabine dose of 100 mg/m^2^ IV on days 1 to 10 compared to a higher dose of cytarabine, 3 g/m^2^ twice daily on days 1, 3, 5 and 7 for a total of eight doses, and found higher CR rates with high dose cytarabine 82% vs. 76% (*p* = 0.01); however, this did not translate to improved OS at the end of 6 years HR 0.89 and *p* = 0.06 [11]. Subgroup analysis revealed a 6-year OS of 51.9% compared to 43.3% (*p* = 0.009) in younger patients (15 to 45 years) favoring high-dose cytarabine. Of note, in both Lowenberg’s and Willemze’s trials, only younger patients ≤60 years were included, with a median age of 47 years. Given that real-world patients are older with worse performance status, the perceived benefit of improved relapse-free survival needs to be very cautiously explored, if at all, in the younger population; therefore, the current standard of care for induction therapy in AML uses low-dose infusional cytarabine at 100–200 mg/m^2^ on days 1–7 [12].

### 3.3. Anthracycline Choice and Dose

Anthracyclines are potent topoisomerase-II inhibitors used in a variety of solid and hematologic malignancies and form the mainstay of treatment for AML. Multiple studies have looked at different anthracyclines, including doxorubicin, daunorubicin, idarubicin and mitoxantrone and their various doses. However, daunorubicin and idarubicin remain the most widely used anthracycline drugs in the United States.

#### 3.3.1. Optimizing Daunorubicin Dosing in AML

Löwenberg et al. randomized patients aged 60 and above to either daunorubicin 90 mg/m^2^ or 45 mg/m^2^ for days 1 to 3, combined with a cytarabine 200 mg/m^2^ continuous dose for 7 days [13]. There was a striking difference in CR rates of 64% in the higher dosage group compared to 54% in the 45 mg/m^2^ arm with a *p* = 0.002. This difference did not translate into a difference in OS for the entire cohort but reached significance in the cohort aged 60–65 years with an HR of 0.65 and *p* = 0.007, favoring the 90 mg/m^2^ dose. Importantly, no differences were present in the rates of early or late mortality between the two dose groups. The ECOG group also reported their phase three randomized E1900 trial in 657 patients aged less than 60 years who were similarly randomized to daunorubicin 90 mg/m^2^ vs. 45 mg/m^2^ days 1 to 3 to be given with cytarabine 100 mg/m^2^ as a continuous dose day 1 to 7 for newly diagnosed AML patients in the same journal issue as the European group [14]. The CR rates were favorable in the higher dose group, 70.6%, compared to 57.3% in the 45 mg/m^2^ dose group (*p* < 0.001) without an increase in deaths (*p* = 0.60). The median OS was better in the higher dose group, 23.7 months vs. 15.7 months in the 45 mg/m^2^ dose group (HR 0.74, *p* = 0.003). During subgroup analysis, it was noted that this survival advantage was driven primarily by patients aged less than 50 years (*p* = 0.004), the favorable (*p* = 0.02) and intermediate cytogenetic group (*p* = 0.004) and that there was no advantage in the older patients (*p* = 0.20) and the unfavorable cytogenetic group (*p* = 0.45). With a longer follow-up median of 80.1 months, results from the E1900 trial demonstrated the survival benefit extending across all cytogenetic groups in patients less than 50 years, including unfavorable cytogenetics group (HR 0.66, *p* = 0.04). There was OS improvement with the higher dose in older patients aged 50–60 years if they had an FLT3-ITD or NPM1 mutation compared to patients who received the standard dose [15].

The French group reported retrospective data comparing daunorubicin 90 mg/m^2^ vs. 60 mg/m^2^; no difference was noted in CR, OS or toxicity between the two groups across all cytogenetic groups [16] with a trend towards improved survival (*p* = 0.07) in the core binding factor (CBF) AML [17]. The UK National Cancer Research Institute reported the results of the AML17 trial in patients across all age groups who received double induction with cytarabine 100 mg/m^2^ twice daily day 1 to 10 and were randomized to daunorubicin 90 mg/m^2^ vs. 60 mg/m^2^ days 1, 3 and 5, followed by a second induction course with daunorubicin 50 mg/m^2^ and cytarabine 100 mg/m^2^ twice daily from days 1 to 8 [18]. They reported no differences in the CR rates and OS rates between the two dose groups and also noted an increased 60-day mortality rate of 10% in the higher dose 90 mg/m^2^ group compared to 5% in the 60 mg/m^2^ group (*p* = 0.001). The results from this study drew criticism that the mandated second induction with daunorubicin 50 mg/m^2,^ irrespective of the remission status, meant that both groups eventually received a higher anthracycline dose as defined by the E1900 trial, causing excess toxicity and counteracting any survival advantage. Seven years later, at the American Society of Hematology conference in 2022, a German group presented the SAL Dauno-Double trial data with the goal of addressing two questions using a two-stage randomization design. Stage I randomized patients to daunorubicin 60 mg/m^2^ vs. 90 mg/m^2,^ and stage II randomized patients to receive a single vs. double induction [19]. Patients were initially randomized to daunorubicin 60 vs. 90 mg/m^2^, followed by a second randomization in good responders defined as patients who achieved less than 5% blasts on the day 15 marrow to a second induction vs. observation. The primary endpoint of the first randomization step was achieving a blast count <5%, defined as a “good response”. Patients who achieved a “good response” are then randomized to single vs. double induction, and the primary endpoint of the second randomization stage is CR/CR with incomplete count recovery after the completion of induction with an assumed non-inferiority margin of 7.5%. A total of 218 patients were randomized to daunorubicin 60 vs. 90 mg/m^2^, and an interim analysis was conducted based on day 15 marrow response. Less than 5% blasts on day 15 marrow were achieved by 42% vs. 49% of patients in the 60 vs. 90 groups, respectively, (*p* = 0.341). Based on these results and pre-planned analyses, the authors concluded the non-inferiority of daunorubicin 60 mg/m^2^ and thereafter, all patients received daunorubicin 60 mg/m^2^. In patients who received the second randomization, the data were very conclusive that a second induction with daunorubicin does not add to survival in patients with a hypoplastic marrow at day 15 (*p* = 0.914). In AML, CR may be used as a surrogate endpoint for OS; however, day 15 marrow response is yet again a potential surrogate for CR. Experts are questioning the validity of using day 15 marrow response to prove the non-inferiority of one dose level over another. We eagerly await the final publication to better understand the study design and whether this would change daunorubicin dosing recommendations on national guidelines.

#### 3.3.2. Which Anthracycline Is Best in AML?

A large meta-analysis published in 1998 looked at all available trials and found five eligible comparable studies between idarubicin (IDA) and daunorubicin (DNR), who shared their individual data [20]. The complete remission rates for the IDA group were 62.4% compared to 53.2% with daunorubicin (*p* = 0.002), with no difference in early induction failure rates at less than 40 days and an increased late induction failure rate with daunorubicin compared with IDA (28.8% vs. 17.5%) *p* < 0.0001. These results also translated into increased overall survival rates at 5 years, with the IDA group doing better at 13% compared to 9% with daunorubicin, HR 0.86 and *p* = 0.03. However, a deeper dive into the data reveals that the daunorubicin dose in four out of the five studies was 45 mg/m^2^ for 3 days and 50 mg/m^2^ for 3 days in the last one compared to an accumulated IDA dose of 36–40 mg/m^2^, about 12 mg/m^2^ for 3 days. We now know that daunorubicin 45 and idarubicin 12 are not equivalent doses. The ALFA 9801 study in patients aged 50 to 70 years used a cytarabine 200 mg/m^2^ continuous dose from day 1 to 7 combined with either daunorubicin 80 mg/m^2^ for 3 days or IDA 12 mg/m^2^ given for 3 days (IDA3) or 4 days (IDA4) and reported about 150 patients in each arm [21]. The CR rates for DNR were 70%, IDA3 was 83%, and IDA4 was 78% (*p* = 0.04), with no difference in OS between the three arms (*p* = 0.19). All three arms had comparable toxicity profiles, with more mucositis reported with increasing doses of idarubicin compared to DNR. The long-term outcomes of this study were combined with data from another French trial, the ALFA 9803, that used a cytarabine continuous 200 mg/m^2^ dose for 7 days either with DNR 45 mg/m^2^ for 4 days or idarubicin 9 mg/m^2^ for 4 days and was reported by Gardin et al. [22]. In a total of 727 patients aged over 50 years, they reported a CR rate in the IDA arm, 69%, compared to 61% with DNR (*p* = 0.029), with no difference in survival noted (*p* = 0.13). They also provided a cure rate model, which showed better rates of cure with IDA 16.6% vs. DNR 9.8% (HR 0.77, *p* = 0.018). Lee et al. conducted a randomized control trial in South Korea where newly diagnosed AML patients were randomized to receive idarubicin 12 mg/m^2^/day or daunorubicin 90 mg/m^2^/day on days 1–3, given with a continuous dose of cytarabine 200 mg/m^2^ days 1 to 7 [23]. Reinduction was allowed with a 5 + 2 course with idarubicin 8 mg/m^2^ and daunorubicin 45 mg/m^2^ for patients who had persistent leukemia in the day 14 marrow. A total of 299 patients were randomized, with 149 receiving idarubicin and 150 patients receiving daunorubicin. CR rates were no different in patients requiring a single course of induction (IDA vs. DNR, 71.1% vs. 66.7%, *p* = 0.403) and second course of induction (IDA vs. DNR, 50% vs. 36.4%, *p* = 0.283). There was no difference in OS noted between the arms at the end of the 4-year follow-up period, *p* = 0.756. Another interesting finding from the subgroup analysis was that when FLT3-ITD mutation was present, OS at 4 years was statistically significantly better in the arm that received DNR compared to IDA (61.9% vs. 30.8%, *p* = 0.946). Finally, none of these studies demonstrate a difference in survival when either IDA or DNR is used, and the choice between the two agents is often dictated by institutional preference and drug availability.

## 4. Building on the 7 + 3 Backbone

### 4.1. Gemtuzumab Ozogamicin (GO)

This monoclonal antibody directed against CD33 linked to an antitumor antibiotic, calicheamicin, was originally granted regulatory approval by the Food and Drug Administration (FDA) in 2000 [24] for AML patients over 60 years after their first relapse. A dose of 9 mg/m^2^ was given on day 1 and repeated on day 15 based on the results of three open-label trials showing 30% CR rates [25]. However, when the interim analysis of the confirmatory phase III trial [26] failed to show efficacy with the addition of gemtuzumab 6 mg/m^2^ on day 4 to standard intensive chemotherapy DNR 45 mg/m^2^ on day 1 to 3 and cytarabine 100 mg/m^2^ continuous dose day 1 to 7 along with safety signals from prior studies showing increased veno-occlusive disease [27], the drug was voluntarily withdrawn from the market in 2010. Again, seven years later, US Food and Drug Administration (FDA) re-approved GO as the first-line treatment for patients with de novo AML based on the pivotal ALFA 701 clinical trial [28] and a meta-analysis by Hills et al. from individual patient level data from 5 clinical trials [29]. In this trial patients aged 50–70 years were given GO as 3 mg/m^2^ on days 1, 4 and 7 in combination with daunorubicin 60 mg/m^2^ day 1 to 3 and a cytarabine continuous dose 200 mg/m^2^ day 1 to 7, resulting in improved event-free survival. However, no difference in CR rates was seen (*p* = 0.25), though improved 2-year OS was initially shown (*p* = 0.03). Final overall survival in ALFA 701 trial on 30 April 2013 cut-off favored gemtuzumab ozogamicin but was not significant. Of note, only nine patients (3%) in this trial had AML with favorable cytogenetics. There was a concurrent approval in the relapsed refractory (R/R) setting as a single agent based on two trials. In phase III of the AML-19 trial, gemtuzumab was given in R/R AML patients over 60 years as 6 mg/m^2^ on day 1 and 3 mg/m^2^ on day 8, resulting in improved survival by ~6 weeks compared to best supportive care [30] and the second, an earlier phase II trial called MyloFrance-1 which explored a new fractionated dosing given as 3 mg/m^2^ on day 1, 4 and 7 [31]. Multiple meta-analyses have demonstrated that when all data is pooled, the survival benefit is primarily in the group with favorable and, to a lesser degree, intermediate-risk cytogenetics. Hence most societies often recommend upfront use in favorable or intermediate-risk cytogenetics. [29,32] Per the 2022 European Leukemia Net, GO is widely administered on day 1 of induction only and on day 1 in up to 2 cycles of consolidation [33]. A single-center retrospective study from MD Anderson in patients with core binding factor CBF AML shows that FLAG-GO gives a survival advantage over FLAG-IDA (*p* = 0.04), which is more pronounced in patients younger than 60 years. However, we do not have adequate data to make any conclusions regarding how they perform compared to 7 + 3 with GO or higher doses of 7 + 3 using DNR 90 mg/m^2^.

### 4.2. FLT3 Inhibitors

FMS-like tyrosine kinase 3 (*FLT3*) gene mutation is one of the common mutations seen in AML in up to 30% of newly diagnosed cases, recognized as a proliferative signal associated with poor prognosis. The phase 3 RATIFY trial-CALGB10603 reported by Stone et al. led to the FDA approval of a FLT3 inhibitor, midostaurin, the first targeted agent in 2017 after the removal of gemtuzumab in 2010 [34]. Midostaurin 50 mg oral was added on days 8 to 21 to the 7 + 3 induction with daunorubicin 60 mg/m^2^ from day 1 to 3 and a cytarabine 200 mg/m^2^ continuous dose days 1 to 7, and continued during a consolidation with a similar dosing schedule and saw an improvement in CR rates 59% vs. 54%, *p* = 0.045 and median survival 74.7 months vs. 25.6 months [hazard ratio for death, 0.78, one-sided *p* = 0.009]. Anemia, rash, QTc prolongation and diarrhea seemed to be the major side effects noted with the addition of midostaurin. Another *FLT3* inhibitor, quizartinib, was granted priority review by the FDA based on the positive results of the phase III trial QuANTUM-First, which randomized newly diagnosed *FLT3-ITD* positive AML patients aged 18–75 to receive quizartinib 40 mg/day orally from days 8 to 21 vs. placebo in addition to induction with a cytarabine 100 mg/m^2^ continuous dose days 1 to 7 and daunorubicin 60 mg/m^2^ or idarubicin 12 mg/m^2^ days 1 to 3 [35]. There was a 22.4% reduction in the risk of death with quizartinib compared to standard chemotherapy alone (HR = 0.776; 2-sided *p* = 0.032) in patients with newly diagnosed FLT3-ITD positive AML. The median OS was more than double at 31.9 months for patients receiving quizartinib (95% CI: 21.0-NE) compared to 15.1 months for patients receiving chemotherapy (95% CI: 13.2–26.2). The HOVON 156 AML trial is another phase III trial currently in the last stages, having accrued 738 of the 768 needed and is currently on a brief HOLD and will shed light on the efficacy of upfront gilteritinib another FLT3 inhibitor that is being compared to midostaurin when added to 7 + 3 induction chemotherapy.

### 4.3. Lomustine

This alkylating agent was evaluated by French groups in three different phase III studies in AML patients aged more than 60 years for induction with idarubicin 8 mg/m^2^ day 1 to 5 and a cytarabine 100 mg/m^2^ continuous dose day 1 to 7 with lomustine 200 mg/m^2^ and without showed a survival benefit 12.7 months compared to 8.7 months (*p* = 0.004) [36]. A recent posthoc analysis of one of these studies shows that the survival benefit is in elderly patients with RUNX1, ASXL1, TP53 and FLT-ITD^high^/NPM1^WT^ mutations [37]. However, there are no studies that have evaluated this agent in the United States.

## 5. Liposomal Formulation of Cytarabine and Daunorubicin CPX-351

CPX-351, now better known as VYXEOS, is a liposomal encapsulation of cytarabine and daunorubicin in a fixed molar ratio of 5:1. Each VYXEOS unit contains 1 mg of cytarabine and 0.44 mg of daunorubicin. The phase III trial by Lancet et al. [38], which led to its FDA approval in August 2017, was conducted in 309 AML patients aged 60 to 75 years. Patients randomized had to have a diagnosis of therapy-related AML, AML with a history of MDS with or without prior hypomethylating agents, AML with a history of chronic myelomonocytic leukemia and de novo AML with MDS-related changes (AML-MRC). Standard induction chemotherapy with a cytarabine 100 mg/m^2^ continuous dose for days 1 to 7 and daunorubicin 60 mg/m^2^ on days 1 to 3 were compared to VYXEOS 100 units/m^2^ followed by a second induction in patients who did not achieve a hypoplastic marrow by day 14 with 5 + 2 vs. VYXEOS 65 units/m^2^. The overall survival improved using VYXEOS with a median OS of 9.5 months vs. standard 7 + 3 induction with a median OS of 5.6 months (HR 0.69, *p* = 0.003). Improved CR rates after the first induction cycle were seen across all age groups and AML subtypes; however, in patients who had already declared themselves chemo-resistant post-initial induction, a second induction with CPX-351 did not lead to better CR rates. The major toxicity noted was delayed count recovery with VYXEOS, a median of 35 days vs. 29 days; however, this did not translate into an increased rate of infection in the trial. More bleeding events were noted with CPX-351. It is believed that along with the synergistic effect of the 5:1 ratio, the liposomal formulation is able to bypass the drug efflux pumps and thereby resistance due to P-glycoproteins and also circumvent rapid denaturation by cytarabine deaminase enzyme in the body leading to better efficacy. However, the biggest barrier for VYXEOS is its cost effectiveness in an already stressed healthcare system [39].

## 6. Lower Intensity Induction for Older Patients with AML

**Figure 2:**
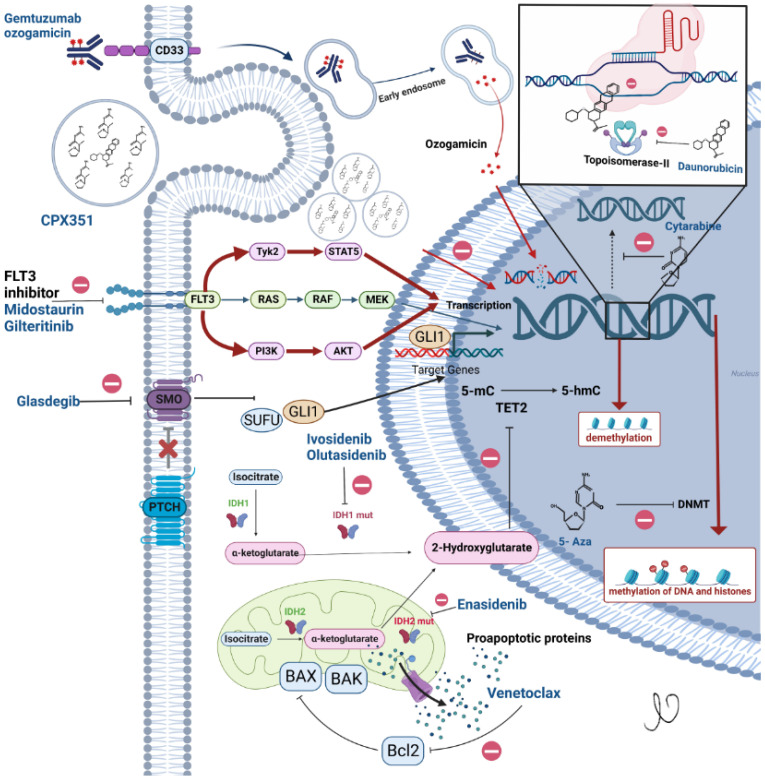
Current FDA approved medications in acute myeloid leukemia and their mechanism of action.

For more than 30 years after 7 + 3 chemotherapy became standard practice, most trials in AML did not include older patients. While some did include older patients, the regimens used were truly palliative in nature. Though different approaches are now available for upfront treatment in this group, the unclear question that still remains is defining the individual’s ineligibility for intensive chemotherapy. Given the increased mortality seen among patients greater than 75 years and overall complications, we generally avoid intensive chemo in this group, but it may be considered on a case-by-case basis up until 79 years of age. Patients with multiple medical comorbidities where the expected mortality with intensive induction is more than 10% may also be offered a less intensive approach to treatment. Additionally, some disease characteristics, such as having a *TP53*-positive AML, may favor a less intensive approach due to the lower chances of CR with intensive chemotherapy. Most leukemia experts still use the “oculometer”, as described by Dr. Kantarjian, which involves making a visual assessment of the patient’s performance status to decide between intensive chemotherapy vs. lower intensity therapy modalities; however, the American Society of Hematology set forth guidelines for the management of older adults with AML that may aid with decision making [40]. Figure 2 shows the FDA-approved agents in AML and their mechanism of action.

### 6.1. Low Dose Cytarabine

Tilly et al. conducted a randomized trial in 87 French AML patients over the age of 65 to receive either low-dose cytarabine 20 mg/m^2^ for 21 days or intensive chemotherapy with rubidazone 100 mg/m^2^ for 4 days and cytarabine 200 mg/m^2^ for 7 days and found no difference in median survival between the two groups (*p* > 0.12), despite CR rates in the intensive group being over 50% compared to 32% in low dose cytarabine arm (*p* < 0.001) [41]. Increased early mortality due to the intensive induction due to secondary infection in the setting of prolonged cytopenias was the likely reason for similar overall survival in both groups. The AML 14 trial by the UK National Cancer Research Institute comprised of 217 patients who were randomized between low-dose cytarabine 20 mg/m^2^ subcutaneously (subQ) given twice daily for 10 days vs. hydroxyurea dose adjusted to keep white counts below 10 × 10^9^/L [42]. CR rates and OS were found to be better compared to hydrea across all age groups. These studies form the basis to use low-dose cytarabine for older adults with AML.

### 6.2. DNA Methyltransferase (DNMT) Inhibitors

Approval of decitabine, a hypomethylating agent that inhibits DNA methyltransferase, as a treatment modality in MDS led to early phase studies in AML, paving the way to a multicentered randomized phase III trial in newly diagnosed AML [43]. A total of 485 patients were randomized to decitabine 20 mg/m^2^ IV for 5 days vs. preferred treatment, which could be cytarabine 20 mg/m^2^ subQ daily for 10 days every 4 weeks or supportive care or patient/physician preferred regimen. Though the total remission rates were significantly better in the decitabine group, 17.8% vs. 7.8% (*p* = 0.001), this did not translate into an overall survival improvement. Importantly, no increase in treatment-related deaths was noted between the two groups. An international phase III trial of azacitidine 75 mg/m^2^ given subQ daily for 7 days and repeated every 28 days was randomized in a similar fashion as the previous study and saw improvement in median overall survival 12.1 months vs. 6.9 months in the prespecified sensitivity analysis (*p* = 0.019) without signals of increased side effects or drug-related mortality [44].

## 7. FDA Approved Lower-Intensity Upfront Combinations

### 7.1. Venetoclax

Studies demonstrating increased expression of BCL-2 in Leukemia stem cells and its ability to induce differential apoptosis compared to HSC with inhibition of BCL-2 in Acute Myeloid Leukemia [45] led to further studies that evaluated different BCL-2 inhibitors in AML. Venetoclax, one such BCL-2 inhibitor, was a major improvement from previous molecules as it did not have the concurrent BCL-X_L_ inhibition that led to significant hematologic toxicities. Following its success in other leukemias, though venetoclax only showed modest single-agent activity in AML, strong preclinical data suggesting increased expression of BCL-2 and MCL1 in AML and the possibility of indirectly targeting MCL1 with cytotoxic drugs kept the researchers going [46]. These efforts bore fruit in the form of two practice-changing studies combining venetoclax with low-dose cytarabine [47] (VIALE-C) and the second with azacitidine in the VIALE-A trial [48], which led to an accelerated FDA approval for both combinations in November 2018 followed by regular approval in October 2020.

#### Venetoclax with Low Dose Cytarabine

A clear example of bench-to-bedside success was the continued faith demonstrated by researchers in the combination of venetoclax with cytotoxic agents. This phase III double-blind placebo-controlled study was the first major publication that later opened the pandora of choices for induction treatment in the elderly [47]. A total of 143 patients in the study arm received oral venetoclax 600 mg in a dose ramp-up fashion with low dose cytarabine 20 mg/m^2^ subQ given days 1 to 10 achieved CR/CRi rates in almost half the study arm compared to 13% in the placebo arm (*p* < 0.001). After an additional 6 months from the originally planned analysis, even a survival advantage was confirmed in this arm at 8.4 months vs. 4.1 months (*p* = 0.04). With similar rates of treatment discontinuation, this regimen was well tolerated by elderly and unfit patients and led to its FDA approval for upfront therapy, creating a new option for a large proportion of these patients who had prior HMA exposure.

### 7.2. Venetoclax with DNMT Inhibitors

#### 7.2.1. Venetoclax with Azacitidine

The VIALE-A study was a phase III placebo-controlled effort where azacitidine 75 mg/m^2^ was given daily from days 1 to 7, combined with venetoclax oral pills 400 mg once daily or placebo, in newly diagnosed AML patients over the age of 18 years who were deemed ineligible for intensive chemotherapy based on predefined criteria which included age ≥ 75 years or younger patients with at least one comorbidity or an ECOG performance status of two or three. A total of 433 patients with a median age of 76 were randomized across 27 countries and showed a median overall survival of 14.7 months in the venetoclax/azacitidine group compared to 9.6 months in the azacitidine/placebo arm, HR for death was 0.66 and *p* < 0.001. Most importantly, unlike the azacitidine-only arm, the responses with the combination were rapid, and nearly half the responses occurred before the second cycle was given, making this a true induction regimen. Molecular analysis showed that the subgroup with IDH mutations had the most benefit. Hematologic toxicities and increased time for count recovery were the main issues the venetoclax group in the study encountered. Now physicians are more familiar with these agents and have generously titrated the number of doses of the venetoclax based on individual patient marrow recovery periods and have also employed longer cycles with the addition of granulocyte colony-stimulating factor (for patients in remission), which have led to better real-world tolerability of this regimen.

#### 7.2.2. Venetoclax with Decitabine

The FDA approval for this combination in patients aged > 75 years was based on the results of the phase Ib dose escalation and expansion study that included 73 of the 145 patients who received venetoclax in combination with decitabine 20 mg/m^2^ IV on days 1 to 5 of each 28-day cycle, of which 27 were above 75 years old [49]. Given the very similar safety profile noted between azacitidine and decitabine, a phase III trial for this combination will not be conducted, given the success of the VIALE-A study. However, there are now real-world propensity score-matched analyses of patients who received decitabine, with or without venetoclax, showing superior survival with the combination compared to decitabine alone at 13.4 months vs. 8.3 months, *p* = 0.01 without additional safety signals, even in the population that received hematopoietic stem cell transplant (HSCT) [50].

### 7.3. Glasdegib with Low Dose Cytarabine

Studies to better understand chemoresistance mechanisms in AML showed that there is activation and increased signaling from the hedgehog pathway in multi-drug resistant cell lines of AML compared to de novo AML [51]. Following the results of an open-label phase II randomized BRIGHT trial where low dose cytarabine 20 mg subQ twice daily days 1–10 was administered with glasdegib 100 mg PO daily vs. placebo, that showed a survival advantage with the combination 8.8 months vs. 4.9 months, *p* = 0.0004 reported at 80% confidence interval, the FDA approved the above combination in November 2018 for patients aged > 75 years [52]. Patients in the experimental arm had a higher incidence of pneumonia, dysgeusia and muscle spasms. Updated results from this predominantly septuagenarian population have continued to show significantly improved survival across all cytogenetic groups, including poor risk cytogenetics without the emergence of new adverse side effects [53].

### 7.4. Isocitrate Dehydrogenase (IDH) Inhibitors with Azacitidine

Ivosidenib, the IDH1 inhibitor, was approved by the FDA originally in an R/R setting and for upfront use in intensive chemo-ineligible patients as a single agent. A phase III double-blind trial randomized 295 patients with newly diagnosed AML who were ineligible for intensive chemotherapy to receive azacitidine 75 mg/m^2^ daily on days 1 to 7 with ivosidenib PO 500 mg daily vs. placebo for 28-day cycles. The results demonstrated improvement in the primary endpoint of EFS. Additionally, overall survival was prolonged with the combination with a median OS of 24 months compared to 7.9 months (*p* = 0.001) [54]. In more than half the patients, 53% achieved CR +CRi in contrast to 18% in the azacitidine-only arm (*p* < 0.001). The combination had higher bleeding events 41% vs. 29% but led to an overall improvement in health-related quality of life and increased transfusion independence. With the FDA approval of this combination in May 2022, there arises a new question regarding the sequencing of these combinations [venetoclax vs. ivosidenib] with azacitidine. However, a similar phase 2 trial of enasidenib, an IDH2 inhibitor with azacitidine, did not show any OS benefit in newly diagnosed AML patients [55].

## 8. Relapsed/Refractory AML

Patients who fail to achieve a CR post two cycles of intensive induction regimen are classified as primary refractory disease and are the fate of 20 to 30% of all newly diagnosed AML. However, there is still a large group who are unable to receive intensive treatment in the first place. Relapsed AML is even more challenging owing to increased chemoresistance and an already beaten-up protoplasm from the prior therapies. A small fraction of patients who have relapsed late after intensive chemotherapy and continue to be fit can be considered for further intensive chemotherapy. The only curative option in the relapsed setting continues to be hematopoietic stem cell transplantation [56]. In patients who are unfit for transplant or intensive chemotherapy, targeted agents have emerged which give multiple therapeutic options.

### 8.1. Hematopoietic Stem Cell Transplant (HSCT)

Based on multiple retrospective trials and metanalysis, it is evident that the best outcome in relapsed AML is achieved if they are able to receive a stem cell transplant in CR. The concept of minimal residual disease (MRD) is a new one as far as AML is concerned and is garnering considerable attention to better define the high relapse risk population and if MRD translates to survival advantage if achieved prior to HSCT. Guidelines from the European Leukemia Net were published to help standardize recommendations on methods of testing and the clinical impact of MRD results in AML [57]. HSCT is a complicated process with multiple variables, including donor selection, conditioning regimens and graft vs. host disease (GVHD) prophylaxis, which are way beyond the scope of this review. Recently at the plenary session of ASH 2022, a German group presented their phase III ASAP trial comprising patients with relapsed AML or patients who did not achieve remission post-one cycle of intensive chemotherapy and were eligible for intensive chemo and allogeneic SCT. They were randomized to receive intensive chemotherapy with a remission induction strategy followed by transplant vs. a wait-and-watch strategy to receive early transplant with the use of a disease control regimen as needed [58]. Surprisingly, no difference has been noted between the two groups in the primary endpoint, CR at day 56 post-transplant and the secondary endpoint of overall survival, questioning a central dogma in AML treatment regarding the role of intensive salvage chemotherapy in relapse. Most leukemia specialists are eagerly awaiting the complete results and follow-up on this possibly practice-changing trial.

### 8.2. Targeted Therapy Approved in Relapsed/Refractory AML

#### 8.2.1. Isocitrate Dehydrogenase Inhibitors

Studies using whole exome sequencing led to the discovery of multiple recurrently mutated genes that helped establish the landscape in AML and opened up opportunities for targeted therapies [4]. One such frequently mutated epigenetic regulator is isocitrate dehydrogenase (IDH) mutations. There are three commonly recognized IDH genes, IDH1, IDH2 and IDH3. These gene transcripts make IDH1 and IDH2 enzymes which catalyze the conversion of isocitrate to alpha-ketoglutarate in the cytoplasm and mitochondria, respectively. Mutations in IDH1 account for about 8% of all mutations in AML, while IDH2 constitutes 12%. Mutated IDH enzymes attain neomorphic activity and convert isocitrate to 2-hydroxyglutarate, which affects DNA methylation leading to a block in cellular differentiation. Translational work using small molecule inhibitors of IDH led to the first-ever targeted therapy approval in relapsed AML.

#### 8.2.2. Enasidenib-IDH2 Inhibitor

AG-221, now popularly known as enasidenib, was the first-in-class oral inhibitor of IDH2 mutations. Stein and colleagues [59] reported the results of their early phase dose escalation study with this novel agent in 239 patients with IDH2 mutated R/R AML, and was soon followed by its FDA approval the same year in August 2017. Enasidenib 100 mg given daily elicited an overall response rate (ORR) of 40.3%, along with a CR noted in ~20% of the patients. Being a non-cytotoxic agent, enasidenib was well tolerated, and the major adverse effects noted were indirect hyperbilirubinemia, tumor lysis syndrome and “differentiation syndrome”. Differentiation syndrome, seen in about 14% of patients, has a high mortality risk without early recognition and treatment, prompting FDA to issue a black box warning on the label. The blasts rapidly proliferate and differentiate into mature neutrophils under the influence of enasidenib, leading to infiltration into tissues causing pulmonary infiltrations, hypoxia, pericardial effusions and fevers. We now recognize that this can happen as late as 4–5 months into treatment and respond well to oral steroids such as dexamethasone 10 mg twice daily. However, the phase III randomized trial of IDH2 inhibitor in R/R AML failed to show an improvement in overall survival compared to conventional regimens (*p* = 0.2), despite a significant improvement in event-free survival likely due to the substantial number of patients who dropped out from the non-enasidenib arm and received enasidenib as a subsequent therapy [60].

#### 8.2.3. Ivosidenib—IDH1 Inhibitor

IDH1 mutations are less frequent in AML compared to IDH2; however, the more durable responses to oral inhibitor AG-120, now called ivosidenib, led to its FDA approval initially for R/R AML with IDH1 mutations in July 2018, soon followed by approval for newly diagnosed AML patients who were unable to obtain intensive chemotherapy by May 2019. The early phase dose escalation and expansion study reported by Dinardo et al. [61] involved 268 patients and recommended a phase II dose of 500 mg daily. They reported CR of 21.6% and ORR of 42% in this R/R cohort. The median OS of the entire cohort was 8.8 months, with an OS of 18 months in patients who attained CR/CR with partial hematologic recovery. About 21% of the patients had mutational clearance by PCR. Data from 34 newly diagnosed AML patients with IDH1 mutations, part of the initial phase I study, was reported separately and showed a CR + CRh rate of 42.4% in the upfront setting, with induction death less than 3%. Most adverse events were gastrointestinal, with diarrhea reported in 53%, along with nausea in 38%. A unique side effect was the QTc prolongation seen in 8% of patients and differentiation syndrome in about 4%. A follow-up study that looked at co-occurring mutations found that JAK2 mutations predicted response while RAS mutations correlated with resistance [62]. Another important finding from the same paper was that there was no association between the variant allele frequency (VAF) of IDH mutations in the original sample and response to ivosidenib.

#### 8.2.4. Olutasidenib—IDH1 Inhibitor

FT-2102, now marketed as olutasidenib, is a new selective oral IDH1 inhibitor with irreversible inhibition compared to the reversible inhibition by ivosidenib [63]. Data from Study 2102-HEM-101 was initially presented at the American Society of Hematology 2022 meeting and led to the FDA approval of olutasidenib in December 2022 for relapsed refractory AML with IDH1 mutation. An updated phase I data published recently reports 78 patients who received olutasidenib in a dose escalation manner as monotherapy n = 32 or combination with azacitidine 75 mg/m^2^ IV days 1 to 7 of a 28-day cycle and reported the phase II dose as 150 mg PO twice daily [12]. An ORR of 41% with monotherapy and 46% was seen in combination with azacitidine in the R/R population. Safety signals were similar to the prior IDH inhibitor, and common adverse events included nausea, vomiting, diarrhea, thrombocytopenia and febrile neutropenia. Unique side effects included differentiation syndrome (13%), QTc prolongation 7% and grade III or higher liver function tests in 13% of patients. The percentage of patients receiving prior IDH1 inhibitors in the study is not clear and may help determine the sequence of these small molecule inhibitors.

#### 8.2.5. FLT3 Inhibitor

FLT3 is one of the most frequently mutated genes seen in de novo AML and portends poor survival due to the challenges presented by the proliferative phenotype seen as a result of this mutation and subsequent higher relapse rates. It is now accepted that FLT3 mutations in de novo AML are generally polyclonal; hence broader multi-kinase FLT3 inhibitors such as midostaurin and sorafenib have higher efficacy compared to relapsed AML where a FLT3 subclone gains selective advantage and responds better to more selective inhibitors such as gilteritinib and quizartinib [64]. The ADMIRAL study that led to the FDA approval of the first FLT3 inhibitor, gilteritinib, in November 2018 was conducted across 14 countries and involved 371 patients with relapsed or primary refractory AML [65]. In this phase III trial, patients with confirmed FLT3 ITD or TKD mutations were randomized in a 2:1 ratio favoring the study drug to receive gilteritinib 120 mg PO daily in comparison to a salvage chemo regimen selected by the local investigator. Patients who received gilteritinib had a survival advantage with a median OS of 9.3 months compared to 5.6 months in the chemotherapy group, *p* = 0.007. Gilteritinib was well tolerated, and besides hematologic toxicities, the major side effects noted were diarrhea, increased liver function tests and QTc prolongation. Follow-up results published from the ADMIRAL study show continued survival advantage without the emergence of new adverse events [66]. However, a major concern is that most study patients had not received upfront midostaurin for induction and hence changes to resistance patterns and efficacy in relapsed FLT3 disease with previous FLT3 therapy are unknown. A subgroup analysis of patients with prior midostaurin from the ADMIRAL study who received gilteritinib at relapse was too small to draw conclusions [67].

## 9. Post Remission Therapy (Aka Consolidation Therapy)

The pivotal CALGB study published in 1994 established high-dose cytarabine consolidation as a standard of care post-induction remission in AML patients [68]. After a standard 7 + 3 induction with a continuous dose of cytarabine 200 mg/m^2^ and daunorubicin 45 mg/m^2^, patients were randomized to receive cytarabine 100 mg/m^2^ vs. 400 mg/m^2^ and 3 g/m^2^ twice daily on days 1, 3 and 5 for four cycles. Patients were stratified by age as <40 years, 40–60 years and >60 years and showed OS benefit in patients younger than 60 years with the higher doses of cytarabine. The HR for OS was 0.78 in the 400 mg/m^2^ dose and 0.74 in the 3 g/m^2^ dose, *p* = 0.04. Serious central nervous system toxicity was seen in the high-dose group at 12% compared to none in the other two groups; hence, randomization to the high-dose group in patients aged more than 60 years was discontinued in the middle of the study period. These patients also received four monthly maintenance doses of cytarabine 100 mg/m^2^ and daunorubicin 45 mg/m^2^ given as a 5 + 1 regimen post consolidation which was omitted in later studies due to poor tolerability. Given the small efficacy difference between the 400 mg/m^2^ dosing compared to the 3 g/m^2^ dosing of cytarabine, clinicians wondered if an intermediate dose of cytarabine could provide all benefits and avoid CNS toxicities. The HOVON study had popularized the intermediate dosing, but given the double induction received by all patients, it was not easy to draw clear results [10]. A complex multi-arm study of the AML-15 conducted by the Medical Research Council of the UK is the best evidence we have for comparison between the cytarabine 1.5 g/m^2^ dosing vs. 3 g/m^2^ dosing [69]. No difference in survival was seen between the two groups, 67% vs. 57% in the 3 g/m^2^ vs. 1.5 g/m^2^ groups (*p* = 0.8), with an improved side effect profile with the lower dose. Intermediate doses are now adopted in patients over the age of 60 years; however, as retrospective studies have shown an increased risk of relapse in younger patients with good–risk disease, many experts and NCCN recommend higher dosing in younger patients. A condensed consolidation regimen with cytarabine given on days 1, 2 and 3 was compared to the standard day 1, 3 and 5 regimen with G-CSF given in both arms, showed no difference in survival and is a better alternative option to consider given improved neutrophil recovery times and decreased infection rates with the day 1, 2 and 3 regimens [70]. This opens a possibly more convenient and shorter treatment cycle for patients.

## 10. Maintenance Therapy

Unlike solid malignancies, the concept of maintenance therapy was novel in AML. The advances in multiparametric flow cytometry and the ability to detect minimal residual disease by PCR techniques have probably influenced researchers to explore the use of maintenance therapy. The HOVON 97 study in 116 older adults with AML, aged > 60 years, who were not eligible for transplant and were in CR/CRi post induction were randomized to azacitidine maintenance 50 mg/m^2^ injections on days 1 to 5 of 28-day cycles vs. observation reported improvement in DFS without a difference in OS [71]. The QUAZAR AML-001 trial is the first positive trial in AML showing improved survival with maintenance oral azacitidine [72]. In this phase III double-blind placebo-controlled trial conducted across 23 countries, AML patients over the age of 55 who were in CR/CRi, not candidates for allogeneic SCT, were given oral azacitidine 300 mg daily for days 1 to 14 of a 28-day cycle compared to placebo. In 472 patients, there was an improvement in median OS in the experimental arm at 24.7 months compared to 14.8 months with a placebo (*p* < 0.001). Even though increased neutropenia and GI side effects were noticed with oral maintenance, the overall quality of life was no different between the groups. Subgroup analysis demonstrates that primarily patients who received less than three cycles of consolidation with cytarabine drove the survival advantage. Oral azacitidine was approved by the FDA in September 2020 for maintenance in patients who were unable to complete consolidation therapy.

### Maintenance Therapy Post Allogenic Transplant

The only subgroup that has evidence to support the use of post-transplant maintenance is the FLT3-mutated AML. In the SORMAIN phase II study in FLT3-ITD mutated AML, 83 patients in CR post an allogenic SCT were randomized to sorafenib vs. placebo for a 2-year period [73]. The relapse-free survival was 85% in favor of sorafenib compared to 53.3%, *p* = 0.002, and after a median follow-up of 55.1 months, even though median OS was not reached in both groups, the estimated survival after 2 years in the sorafenib group was 90.5% compared to 66.2 % in the placebo group (*p* = 0.007). Sorafenib was well tolerated, with no major side effects noted, and a phase III trial will be needed to shed further light on the trend seen towards increased GVHD among sorafenib recipients. Based on these results, the NCCN has recommended the use of sorafenib; however, there is no FDA approval for the same. The MORPHO clinical trial was a phase III trial conducted by the blood and marrow transplant clinical trials network (BMT-CTN) evaluating gilteritinib as a maintenance therapy following allogeneic HSCT for patients with FLT3-ITD mutated AML. They announced top-line results with the study not meeting its predefined primary endpoint of RFS for patients treated with gilteritinib compared to placebo. Therefore, we do not have strong evidence supporting post-transplant maintenance at this time. Table 1 lists the commonly used regimens, specifically the drugs, routes, and schedules.

## 11. Conclusions

We have come a long way over the past decade with multiple new agents approved for use in adults with Acute Myeloid Leukemia. The clinical advances and new combination strategies allow for more treatment options. Multiple newer agents are in clinical and preclinical developmental stages for the treatment of this heterogeneous disease. Patients with AML should receive AML-directed therapies over the best supportive care due to the improvement in patient outcomes with treatment. As AML is a disease of the older population, increased frailty seen in this patient population makes the development of newer agents challenging as the medications need to not only be efficacious but also be very well tolerated. There is excitement and hope for the future of patients with AML. “We must accept finite disappointment but never lose infinite hope”, Martin Luther King.

## Figures and Tables

**Table 1:** Common regimens used in acute myeloid leukemia.

Induction Regimens for Newly Diagnosed Acute Myeloid Leukemia (AML)	
**Drugs**	Schedules
“7 + 3”	Anthracycline IV × 3 days Cytarabine IV continuous dose 100–200 mg/m^2^ Day 1–7
“5 + 2”	Anthracycline IV × 2 days Cytarabine IV continuous dose 100–200 mg/m^2^ Day 1–5
Vyxeos	(7 + 3) Vyxeos 100 units/m^2^ IV on Days 1, 3 and 5 (5 + 2) Vyxeos 65 units/m^2^ IV on Days 1 and 3
Gemtuzumab Ozogamicin with 7 + 3	Gemtuzumab 3 mg/m^2^ on Days 1, 4 and 7 “7 + 3”
Gemtuzumab Ozogamicin	Gemtuzumab 6 mg/m^2^ on Day 1 and 3 mg/m^2^ on Day 8 Cycle 1 Followed by up to 8 doses of 2 mg/m^2^ Day 1 every 28 days
Midostaurin with Daunorubicin and Cytarabine	DAUNORUBICIN 60 mg/m^2^ IV day 1–3 Midostaurin 50 mg BID PO Days 8–21 Cytarabine 200 mg/m^2^ IV Day 1–7
Venetoclax + Azacitidine	Azacitidine 75 mg/m^2^ IV or subQ Day 1–7 every 28 days Venetoclax 400 mg PO once daily Day 1–28. (Dose ramped up as 100 mg on Day 1, 200 mg on Day 2 and 400 mg on Day 3 during cycle 1)
Venetoclax + Low dose cytarabine	Low dose cytarabine 20 mg/m^2^ SubQ Day 1–10 every 28 days Venetoclax 600 mg PO once daily Days 1–28 (Dose ramped up as 100 mg on Day 1, 200 mg on Day 2, 400 mg on Day 3 and 600 mg on Day 4)
Glasdegib + Low dose cytarabine	Low dose cytarabine 20 mg BID SubQ Day 1–10 every 28 days Glasdegib 100 mg PO daily Day 1–28
Ivosidenib + Azacitidine	Azacitidine 75 mg/m^2^ subQ Days 1–7 every 28 days Ivosidenib 500 mg PO once daily Day 1–28.
**Regimens for R/R AML**	
Gemtuzumab	Gemtuzumab 3 mg/m^2^ on Days 1, 4 and 7
Enasidenib	Enasidenib 100 mg PO daily
Ivosidenib	Ivosidenib 500 mg PO daily
Olutasidenib	Olutasidenib 150 mg PO BID
Gilteritinib	Gilteritinib 120 mg PO daily
**Post Remission Therapy (Consolidation)**	
Cytarabine	Cytarabine 1.5–3 g/m^2^ given BID on Days 1, 3 and 5 [total 6 doses]
Gemtuzumab + cytarabine	Gemtuzumab 3 g/m^2^ on Day 1 Cytarabine 1.5–3 g/m^2^ given BID on Day 1, 3 and 5
**Maintenance**	
Oral azacitidine	Azacitidine 300 mg PO daily Day 1–14 every 28 days

## Data Availability

Data sharing is not applicable to this article as no new datasets were generated or analyzed during this study. All data included is published online and all reasonable queries will be addressed by the corresponding author upon request.

## References

[1] Madanat Y., Nazha A. (2021). Novel and Investigational Therapies in Acute Myeloid Leukemia. Acute Leukemias.

[2] Papaemmanuil E., Gerstung M., Bullinger L., Gaidzik V.I., Paschka P., Roberts N.D., Potter N.E., Heuser M., Thol F., Bolli N. (2016). Genomic Classification and Prognosis in Acute Myeloid Leukemia. N. Engl. J. Med..

[3] Steensma D.P., Bejar R., Jaiswal S., Lindsley R.C., Sekeres M.A., Hasserjian R.P., Ebert B.L. (2015). Clonal hematopoiesis of indeterminate potential and its distinction from myelodysplastic syndromes. Blood.

[4] Ley T.J., Miller C., Ding L., Raphael B.J., Mungall A.J., Robertson A., Hoadley K., Triche T.J., Laird P.W., Baty J.D. (2013). Genomic and epigenomic landscapes of adult de novo acute myeloid leukemia. N. Engl. J. Med..

[5] Arber D.A., Orazi A., Hasserjian R.P., Borowitz M.J., Calvo K.R., Kvasnicka H.M., Wang S.A., Bagg A., Barbui T., Branford S. (2022). International Consensus Classification of Myeloid Neoplasms and Acute Leukemias: Integrating morphologic, clinical, and genomic data. Blood.

[6] Khoury J.D., Solary E., Abla O., Akkari Y., Alaggio R., Apperley J.F., Bejar R., Berti E., Busque L., Chan J.K.C. (2022). The 5th edition of the World Health Organization Classification of Haematolymphoid Tumours: Myeloid and Histiocytic/Dendritic Neoplasms. Leukemia.

[7] Bishop J.F., Matthews J.P., Young G.A., Szer J., Gillett A., Joshua D., Bradstock K., Enno A., Wolf M.M., Fox R. (1996). A randomized study of high-dose cytarabine in induction in acute myeloid leukemia. Blood.

[8] Weick J.K., Kopecky K.J., Appelbaum F.R., Head D.R., Kingsbury L.L., Balcerzak S.P., Bickers J.N., Hynes H.E., Welborn J.L., Simon S.R. (1996). A randomized investigation of high-dose versus standard-dose cytosine arabinoside with daunorubicin in patients with previously untreated acute myeloid leukemia: A Southwest Oncology Group study. Blood.

[9] Kern W., Estey E.H. (2006). High-dose cytosine arabinoside in the treatment of acute myeloid leukemia: Review of three randomized trials. Cancer.

[10] Löwenberg B., Pabst T., Vellenga E., van Putten W., Schouten H.C., Graux C., Ferrant A., Sonneveld P., Biemond B.J., Gratwohl A. (2011). Cytarabine dose for acute myeloid leukemia. N. Engl. J. Med..

[11] Willemze R., Suciu S., Meloni G., Labar B., Marie J.P., Halkes C.J., Muus P., Mistrik M., Amadori S., Specchia G. (2014). High-dose cytarabine in induction treatment improves the outcome of adult patients younger than age 46 years with acute myeloid leukemia: Results of the EORTC-GIMEMA AML-12 trial. J. Clin. Oncol..

[12] Watts J.M., Baer M.R., Yang J., Prebet T., Lee S., Schiller G.J., Dinner S.N., Pigneux A., Montesinos P., Wang E.S. (2023). Olutasidenib alone or with azacitidine in IDH1-mutated acute myeloid leukaemia and myelodysplastic syndrome: Phase 1 results of a phase 1/2 trial. Lancet Haematol..

[13] Löwenberg B., Ossenkoppele G.J., van Putten W., Schouten H.C., Graux C., Ferrant A., Sonneveld P., Maertens J., Jongen-Lavrencic M., von Lilienfeld-Toal M. (2009). High-dose daunorubicin in older patients with acute myeloid leukemia. N. Engl. J. Med..

[14] Fernandez H.F., Sun Z., Yao X., Litzow M.R., Luger S.M., Paietta E.M., Racevskis J., Dewald G.W., Ketterling R.P., Bennett J.M. (2009). Anthracycline dose intensification in acute myeloid leukemia. N. Engl. J. Med..

[15] Luskin M.R., Lee J.W., Fernandez H.F., Abdel-Wahab O., Bennett J.M., Ketterling R.P., Lazarus H.M., Levine R.L., Litzow M.R., Paietta E.M. (2016). Benefit of high-dose daunorubicin in AML induction extends across cytogenetic and molecular groups. Blood.

[16] Devillier R., Bertoli S., Prébet T., Huguet F., Etienne A., Charbonnier A., Rey J., Delabesse E., D’Incan E., Huynh A. (2015). Comparison of 60 or 90 mg/m^2^ of daunorubicin in induction therapy for acute myeloid leukemia with intermediate or unfavorable cytogenetics. Am. J. Hematol..

[17] Prebet T., Bertoli S., Delaunay J., Pigneux A., Delabesse E., Mozziconacci M.J., Bidet A., Recher C., Vey N. (2014). Anthracycline dose intensification improves molecular response and outcome of patients treated for core binding factor acute myeloid leukemia. Haematologica.

[18] Burnett A.K., Russell N.H., Hills R.K., Kell J., Cavenagh J., Kjeldsen L., McMullin M.F., Cahalin P., Dennis M., Friis L. (2015). A randomized comparison of daunorubicin 90 mg/m2 vs 60 mg/m2 in AML induction: Results from the UK NCRI AML17 trial in 1206 patients. Blood.

[19] Röllig C., Steffen B., Schliemann C., Mikesch J.-H., Alakel N., Herbst R., Haenel M., Noppeney R., Hanoun M., Kaufmann M. (2022). Single Versus Double Induction with “7 + 3” Containing 60 Versus 90 Mg Daunorubicin for Newly Diagnosed AML: Results from the Randomized Controlled SAL Dauno-Double Trial. Blood.

[20] AML Collaborative Group (1998). A systematic collaborative overview of randomized trials comparing idarubicin with daunorubicin (or other anthracyclines) as induction therapy for acute myeloid leukaemia. Br. J. Haematol..

[21] Pautas C., Merabet F., Thomas X., Raffoux E., Gardin C., Corm S., Bourhis J.H., Reman O., Turlure P., Contentin N. (2010). Randomized study of intensified anthracycline doses for induction and recombinant interleukin-2 for maintenance in patients with acute myeloid leukemia age 50 to 70 years: Results of the ALFA-9801 study. J. Clin. Oncol..

[22] Gardin C., Chevret S., Pautas C., Turlure P., Raffoux E., Thomas X., Quesnel B., de Revel T., de Botton S., Gachard N. (2013). Superior long-term outcome with idarubicin compared with high-dose daunorubicin in patients with acute myeloid leukemia age 50 years and older. J. Clin. Oncol..

[23] Lee J.-H., Kim H., Joo Y.-D., Lee W.-S., Bae S.H., Zang D.Y., Kwon J., Kim M.K., Lee J., Lee G.W. (2017). Prospective Randomized Comparison of Idarubicin and High-Dose Daunorubicin in Induction Chemotherapy for Newly Diagnosed Acute Myeloid Leukemia. J. Clin. Oncol..

[24] Bross P.F., Beitz J., Chen G., Chen X.H., Duffy E., Kieffer L., Roy S., Sridhara R., Rahman A., Williams G. (2001). Approval summary: Gemtuzumab ozogamicin in relapsed acute myeloid leukemia. Clin. Cancer Res..

[25] Sievers E.L., Larson R.A., Stadtmauer E.A., Estey E., Löwenberg B., Dombret H., Karanes C., Theobald M., Bennett J.M., Sherman M.L. (2001). Efficacy and safety of gemtuzumab ozogamicin in patients with CD33-positive acute myeloid leukemia in first relapse. J. Clin. Oncol..

[26] Petersdorf S.H., Kopecky K.J., Slovak M., Willman C., Nevill T., Brandwein J., Larson R.A., Erba H.P., Stiff P.J., Stuart R.K. (2013). A phase 3 study of gemtuzumab ozogamicin during induction and postconsolidation therapy in younger patients with acute myeloid leukemia. Blood.

[27] Wadleigh M., Richardson P.G., Zahrieh D., Lee S.J., Cutler C., Ho V., Alyea E.P., Antin J.H., Stone R.M., Soiffer R.J. (2003). Prior gemtuzumab ozogamicin exposure significantly increases the risk of veno-occlusive disease in patients who undergo myeloablative allogeneic stem cell transplantation. Blood.

[28] Castaigne S., Pautas C., Terré C., Raffoux E., Bordessoule D., Bastie J.N., Legrand O., Thomas X., Turlure P., Reman O. (2012). Effect of gemtuzumab ozogamicin on survival of adult patients with de-novo acute myeloid leukaemia (ALFA-0701): A randomised, open-label, phase 3 study. Lancet.

[29] Hills R.K., Castaigne S., Appelbaum F.R., Delaunay J., Petersdorf S., Othus M., Estey E.H., Dombret H., Chevret S., Ifrah N. (2014). Addition of gemtuzumab ozogamicin to induction chemotherapy in adult patients with acute myeloid leukaemia: A meta-analysis of individual patient data from randomised controlled trials. Lancet Oncol..

[30] Amadori S., Suciu S., Selleslag D., Aversa F., Gaidano G., Musso M., Annino L., Venditti A., Voso M.T., Mazzone C. (2016). Gemtuzumab Ozogamicin Versus Best Supportive Care in Older Patients with Newly Diagnosed Acute Myeloid Leukemia Unsuitable for Intensive Chemotherapy: Results of the Randomized Phase III EORTC-GIMEMA AML-19 Trial. J. Clin. Oncol..

[31] Taksin A.L., Legrand O., Raffoux E., de Revel T., Thomas X., Contentin N., Bouabdallah R., Pautas C., Turlure P., Reman O. (2007). High efficacy and safety profile of fractionated doses of Mylotarg as induction therapy in patients with relapsed acute myeloblastic leukemia: A prospective study of the alfa group. Leukemia.

[32] Guo Y., Deng L., Qiao Y., Liu B. (2022). Efficacy and safety of adding gemtuzumab ozogamicin to conventional chemotherapy for adult acute myeloid leukemia: A systematic review and meta-analysis. Hematology.

[33] Döhner H., Wei A.H., Appelbaum F.R., Craddock C., DiNardo C.D., Dombret H., Ebert B.L., Fenaux P., Godley L.A., Hasserjian R.P. (2022). Diagnosis and management of AML in adults: 2022 recommendations from an international expert panel on behalf of the ELN. Blood.

[34] Stone R.M., Mandrekar S.J., Sanford B.L., Laumann K., Geyer S., Bloomfield C.D., Thiede C., Prior T.W., Döhner K., Marcucci G. (2017). Midostaurin plus Chemotherapy for Acute Myeloid Leukemia with a FLT3 Mutation. N. Engl. J. Med..

[35] Erba H., Montesinos P., Vrhovac R., Patkowska E., Kim H.J., Zak P., Wang P.N., Mitov T., Hanyok J., Liu L. (2022). S100: Quizartinib prolonged survival vs placebo plus intensive induction and consolidation therapy followed by single-agent continuation in patients aged 18–75 years with newly diagnosed flt3-itd+ aml. HemaSphere.

[36] Pigneux A., Harousseau J.L., Witz F., Sauvezie M., Bene M.C., Luquet I., Hunault-Berger M., Recher C., Lioure B., Himberlin C. (2010). Addition of lomustine to idarubicin and cytarabine improves the outcome of elderly patients with de novo acute myeloid leukemia: A report from the GOELAMS. J. Clin. Oncol..

[37] Largeaud L., Cornillet-Lefebvre P., Hamel J.-F., Dumas P.-Y., Prade N., Dufrechou S., Plenecassagnes J., Luquet I., Blanchet O., Banos A. (2021). Lomustine is beneficial to older AML with ELN2017 adverse risk profile and intermediate karyotype: A FILO study. Leukemia.

[38] Lancet J.E., Uy G.L., Cortes J.E., Newell L.F., Lin T.L., Ritchie E.K., Stuart R.K., Strickland S.A., Hogge D., Solomon S.R. (2018). CPX-351 (cytarabine and daunorubicin) Liposome for Injection Versus Conventional Cytarabine Plus Daunorubicin in Older Patients with Newly Diagnosed Secondary Acute Myeloid Leukemia. J. Clin. Oncol..

[39] Bewersdorf J.P., Patel K.K., Goshua G., Shallis R.M., Podoltsev N.A., Huntington S.F., Zeidan A.M. (2022). Cost-effectiveness of liposomal cytarabine/daunorubicin in patients with newly diagnosed acute myeloid leukemia. Blood.

[40] Sekeres M.A., Guyatt G., Abel G., Alibhai S., Altman J.K., Buckstein R., Choe H., Desai P., Erba H., Hourigan C.S. (2020). American Society of Hematology 2020 guidelines for treating newly diagnosed acute myeloid leukemia in older adults. Blood Adv..

[41] Tilly H., Castaigne S., Bordessoule D., Casassus P., Le Prisé P.Y., Tertian G., Desablens B., Henry-Amar M., Degos L. (1990). Low-dose cytarabine versus intensive chemotherapy in the treatment of acute nonlymphocytic leukemia in the elderly. J. Clin. Oncol..

[42] Burnett A.K., Milligan D., Prentice A.G., Goldstone A.H., McMullin M.F., Hills R.K., Wheatley K. (2007). A comparison of low-dose cytarabine and hydroxyurea with or without all-trans retinoic acid for acute myeloid leukemia and high-risk myelodysplastic syndrome in patients not considered fit for intensive treatment. Cancer.

[43] Kantarjian H.M., Thomas X.G., Dmoszynska A., Wierzbowska A., Mazur G., Mayer J., Gau J.P., Chou W.C., Buckstein R., Cermak J. (2012). Multicenter, randomized, open-label, phase III trial of decitabine versus patient choice, with physician advice, of either supportive care or low-dose cytarabine for the treatment of older patients with newly diagnosed acute myeloid leukemia. J. Clin. Oncol..

[44] Dombret H., Seymour J.F., Butrym A., Wierzbowska A., Selleslag D., Jang J.H., Kumar R., Cavenagh J., Schuh A.C., Candoni A. (2015). International phase 3 study of azacitidine vs conventional care regimens in older patients with newly diagnosed AML with >30% blasts. Blood.

[45] Lagadinou E.D., Sach A., Callahan K., Rossi R.M., Neering S.J., Minhajuddin M., Ashton J.M., Pei S., Grose V., O’Dwyer K.M. (2013). BCL-2 inhibition targets oxidative phosphorylation and selectively eradicates quiescent human leukemia stem cells. Cell Stem Cell.

[46] Teh T.C., Nguyen N.Y., Moujalled D.M., Segal D., Pomilio G., Rijal S., Jabbour A., Cummins K., Lackovic K., Blombery P. (2018). Enhancing venetoclax activity in acute myeloid leukemia by co-targeting MCL1. Leukemia.

[47] Wei A.H., Montesinos P., Ivanov V., DiNardo C.D., Novak J., Laribi K., Kim I., Stevens D.A., Fiedler W., Pagoni M. (2020). Venetoclax plus LDAC for newly diagnosed AML ineligible for intensive chemotherapy: A phase 3 randomized placebo-controlled trial. Blood.

[48] DiNardo C.D., Jonas B.A., Pullarkat V., Thirman M.J., Garcia J.S., Wei A.H., Konopleva M., Döhner H., Letai A., Fenaux P. (2020). Azacitidine and Venetoclax in Previously Untreated Acute Myeloid Leukemia. N. Engl. J. Med..

[49] DiNardo C.D., Pratz K., Pullarkat V., Jonas B.A., Arellano M., Becker P.S., Frankfurt O., Konopleva M., Wei A.H., Kantarjian H.M. (2019). Venetoclax combined with decitabine or azacitidine in treatment-naive, elderly patients with acute myeloid leukemia. Blood.

[50] Kwag D., Cho B.-S., Bang S.-Y., Lee J.H., Min G.-J., Park S.-S., Park S., Yoon J.-H., Lee S.-E., Eom K.-S. (2022). Venetoclax with decitabine versus decitabine monotherapy in elderly acute myeloid leukemia: A propensity score-matched analysis. Blood Cancer J..

[51] Queiroz K.C., Ruela-de-Sousa R.R., Fuhler G.M., Aberson H.L., Ferreira C.V., Peppelenbosch M.P., Spek C.A. (2010). Hedgehog signaling maintains chemoresistance in myeloid leukemic cells. Oncogene.

[52] Cortes J.E., Heidel F.H., Hellmann A., Fiedler W., Smith B.D., Robak T., Montesinos P., Pollyea D.A., DesJardins P., Ottmann O. (2019). Randomized comparison of low dose cytarabine with or without glasdegib in patients with newly diagnosed acute myeloid leukemia or high-risk myelodysplastic syndrome. Leukemia.

[53] Heuser M., Smith B.D., Fiedler W., Sekeres M.A., Montesinos P., Leber B., Merchant A., Papayannidis C., Pérez-Simón J.A., Hoang C.J. (2021). Clinical benefit of glasdegib plus low-dose cytarabine in patients with de novo and secondary acute myeloid leukemia: Long-term analysis of a phase II randomized trial. Ann. Hematol..

[54] Montesinos P., Recher C., Vives S., Zarzycka E., Wang J., Bertani G., Heuser M., Calado R.T., Schuh A.C., Yeh S.P. (2022). Ivosidenib and Azacitidine in IDH1-Mutated Acute Myeloid Leukemia. N. Engl. J. Med..

[55] DiNardo C.D., Schuh A.C., Stein E.M., Montesinos P., Wei A.H., de Botton S., Zeidan A.M., Fathi A.T., Kantarjian H.M., Bennett J.M. (2021). Enasidenib plus azacitidine versus azacitidine alone in patients with newly diagnosed, mutant-IDH2 acute myeloid leukaemia (AG221-AML-005): A single-arm, phase 1b and randomised, phase 2 trial. Lancet Oncol..

[56] Koreth J., Schlenk R., Kopecky K.J., Honda S., Sierra J., Djulbegovic B.J., Wadleigh M., DeAngelo D.J., Stone R.M., Sakamaki H. (2009). Allogeneic stem cell transplantation for acute myeloid leukemia in first complete remission: Systematic review and meta-analysis of prospective clinical trials. Jama.

[57] Heuser M., Freeman S.D., Ossenkoppele G.J., Buccisano F., Hourigan C.S., Ngai L.L., Tettero J.M., Bachas C., Baer C., Béné M.-C. (2021). 2021 Update on MRD in acute myeloid leukemia: A consensus document from the European LeukemiaNet MRD Working Party. Blood.

[58] Stelljes M., Middeke J.M., Bug G., Wagner E.-M., Mueller L.P., Christoph S., Krause S.W., Bethge W., Jost E., Platzbecker U. (2022). In Patients with Relapsed/Refractory AML Sequential Conditioning and Immediate Allogeneic Stem Cell Transplantation (allo-HCT) Results in Similar Overall and Leukemia-Free Survival Compared to Intensive Remission Induction Chemotherapy Followed By Allo-HCT: Results from the Randomized Phase III ASAP Trial. Blood.

[59] Stein E.M., DiNardo C.D., Pollyea D.A., Fathi A.T., Roboz G.J., Altman J.K., Stone R.M., DeAngelo D.J., Levine R.L., Flinn I.W. (2017). Enasidenib in mutant IDH2 relapsed or refractory acute myeloid leukemia. Blood.

[60] de Botton S., Montesinos P., Schuh A.C., Papayannidis C., Vyas P., Wei A.H., Ommen H., Semochkin S., Kim H.J., Larson R.A. (2023). Enasidenib vs conventional care in older patients with late-stage mutant-IDH2 relapsed/refractory AML: A randomized phase 3 trial. Blood.

[61] DiNardo C.D., Stein E.M., de Botton S., Roboz G.J., Altman J.K., Mims A.S., Swords R., Collins R.H., Mannis G.N., Pollyea D.A. (2018). Durable Remissions with Ivosidenib in IDH1-Mutated Relapsed or Refractory AML. N. Engl. J. Med..

[62] Choe S., Wang H., DiNardo C.D., Stein E.M., de Botton S., Roboz G.J., Altman J.K., Mims A.S., Watts J.M., Pollyea D.A. (2020). Molecular mechanisms mediating relapse following ivosidenib monotherapy in IDH1-mutant relapsed or refractory AML. Blood Adv..

[63] Golub D., Iyengar N., Dogra S., Wong T., Bready D., Tang K., Modrek A.S., Placantonakis D.G. (2019). Mutant Isocitrate Dehydrogenase Inhibitors as Targeted Cancer Therapeutics. Front. Oncol..

[64] Daver N., Schlenk R.F., Russell N.H., Levis M.J. (2019). Targeting FLT3 mutations in AML: Review of current knowledge and evidence. Leukemia.

[65] Perl A.E., Martinelli G., Cortes J.E., Neubauer A., Berman E., Paolini S., Montesinos P., Baer M.R., Larson R.A., Ustun C. (2019). Gilteritinib or Chemotherapy for Relapsed or Refractory FLT3-Mutated AML. N. Engl. J. Med..

[66] Perl A.E., Larson R.A., Podoltsev N.A., Strickland S., Wang E.S., Atallah E., Schiller G.J., Martinelli G., Neubauer A., Sierra J. (2022). Follow-up of patients with R/R FLT3-mutation–positive AML treated with gilteritinib in the phase 3 ADMIRAL trial. Blood.

[67] Perl A.E., Hosono N., Montesinos P., Podoltsev N., Martinelli G., Panoskaltsis N., Recher C., Smith C.C., Levis M.J., Strickland S. (2022). Clinical outcomes in patients with relapsed/refractory FLT3-mutated acute myeloid leukemia treated with gilteritinib who received prior midostaurin or sorafenib. Blood Cancer J..

[68] Mayer R.J., Davis R.B., Schiffer C.A., Berg D.T., Powell B.L., Schulman P., Omura G.A., Moore J.O., McIntyre O.R., Frei E. (1994). Intensive postremission chemotherapy in adults with acute myeloid leukemia. Cancer and Leukemia Group B. N. Engl. J. Med..

[69] Burnett A.K., Russell N.H., Hills R.K., Hunter A.E., Kjeldsen L., Yin J., Gibson B.E., Wheatley K., Milligan D. (2013). Optimization of chemotherapy for younger patients with acute myeloid leukemia: Results of the medical research council AML15 trial. J. Clin. Oncol..

[70] Jaramillo S., Benner A., Krauter J., Martin H., Kindler T., Bentz M., Salih H.R., Held G., Köhne C.H., Götze K. (2017). Condensed versus standard schedule of high-dose cytarabine consolidation therapy with pegfilgrastim growth factor support in acute myeloid leukemia. Blood Cancer J..

[71] Huls G., Chitu D.A., Havelange V., Jongen-Lavrencic M., van de Loosdrecht A.A., Biemond B.J., Sinnige H., Hodossy B., Graux C., Kooy R.V.M. (2019). Azacitidine maintenance after intensive chemotherapy improves DFS in older AML patients. Blood.

[72] Wei A.H., Döhner H., Pocock C., Montesinos P., Afanasyev B., Dombret H., Ravandi F., Sayar H., Jang J.H., Porkka K. (2020). Oral Azacitidine Maintenance Therapy for Acute Myeloid Leukemia in First Remission. N. Engl. J. Med..

[73] Burchert A., Bug G., Fritz L.V., Finke J., Stelljes M., Röllig C., Wollmer E., Wäsch R., Bornhäuser M., Berg T. (2020). Sorafenib Maintenance After Allogeneic Hematopoietic Stem Cell Transplantation for Acute Myeloid Leukemia with FLT3-Internal Tandem Duplication Mutation (SORMAIN). J. Clin. Oncol..

